# Rescue Stenting of Isolated Middle Cerebral Artery (MCA) Dissections (MCAD) with Antithrombogenic Coated Stents and Mono-Antiplatelet Therapy (MAPT)

**DOI:** 10.3390/jcm13154329

**Published:** 2024-07-24

**Authors:** Piotr Pedowski, Jakub Fedorko, Stefan Pataky, Zuzana Gdovinova

**Affiliations:** 1 Department of Radiodiagnostics and Imaging Techniques, P.J. Safarik University and L. Pasteur University Hospital, 04011 Košice, Slovakia; jakub.fedorko@unlp.sk (J.F.); stefan.pataky@unlp.sk (S.P.); 2 Department of Neurology, Faculty of Medicine, P.J. Safarik University and L. Pasteur University Hospital, 04011 Košice, Slovakia; zuzana.gdovinova@upjs.sk

**Keywords:** dissection, MCA, coated stents, MAPT, Cangrelor, stroke, MCAD, pEGASUS, CREDO, antithrombogenic

## Abstract

**Objective:** Acute ischemic stroke (AIS) is a leading cause of death, but isolated middle cerebral artery dissection (MCAD) is rarely reported. The aim of this article is to sum up the current information on this pathology and to explore the technical aspects of its endovascular treatment with emphasis on novel coated, antithrombogenic stents and antiplatelet management. Another part of this article offers our experience with the problematics represented by a small sample group of patients with an MCAD diagnosis who were treated in our center. **Methods:** We conducted literature research and a retrospective review of patients treated for anterior circulation AIS at our comprehensive stroke center from January 2022 to March 2024. The cohort included 16 patients diagnosed with isolated MCAD, 9 received antithrombogenic coated stents, while 7 received bare metal stents. Pharmacological management of coated stents involved the use of Cangrelor for acute antiplatelet therapy, transitioning to oral Ticagrelor. **Results:** Among the 16 patients treated, those with antithrombogenic coated stents showed no major complications and had a lower incidence of intracranial hemorrhage compared to the bare metal stent group. The average National Institutes of Health Stroke Scale (NIHSS) score at discharge improved in both groups. Functional outcomes and mortality rates were slightly better in the coated stent group, but no statistical significance was proven. **Conclusions:** Antithrombogenic coated stents, in conjunction with MAPT, demonstrated a safe and effective option for treating isolated MCAD. These stents offer promising potential for improved outcomes and reduced complications compared to traditional treatments. Further multicentric studies with larger cohorts are recommended to validate these findings.

## 1. Introduction

The middle cerebral artery (MCA) is a critical vessel that supplies blood to large areas of the brain, and its dissection can lead to severe neurological deficits and high morbidity [1,2].

As some retrospective reviews suggest, isolated MCAD can cause approximately 2,4% of all anterior circulation AIS cases [3]. This number is relatively low compared to posterior circulation dissections, which are reported in approximately 12% of posterior circulation AIS patients [4], or ICA dissections, which cause 20–25% of all anterior AIS in age groups under 45 years [5].

Similar to dissections of the posterior circulation and ICA dissections, MCAD is reported to be more prevalent in young adults [2,6]. 

## 2. Pathophysiology

The pathophysiology of MCA dissections is similar to that of any other vessel in the body. The pathological process involves the tearing of the tunica intima and internal part of the tunica media, which creates an entry tear for the blood flow inside the vessel wall. This leads to the separation of its layers and the creation of a false lumen. That can lead to luminal stenosis of the true lumen or its complete occlusion. This disruption in blood flow can lead to significant ischemic damage to the brain [2,6]. 

The initial arterial wall tear can be spontaneous or secondary to trauma, and it may extend longitudinally or radially [2]. In some cases, the dissected vessel may form aneurysms, which pose a risk of rupture and can lead to subarachnoid hemorrhage (SAH) [7].

## 3. Neurological Presentation

Patients with MCAD often present with sudden, severe headaches, which may be described as thunderclap headaches. Other common symptoms include focal neurological deficits, such as hemiparesis, aphasia, and visual disturbances, which are indicative of an ischemic stroke [2]. The onset of symptoms can be acute or subacute, and in some cases, symptoms may fluctuate or progress over time. In severe cases, patients may present with signs of SAH if the dissection leads to an aneurysmal rupture, but this scenario is more common in posterior circulation [3,8].

## 4. Diagnosis

A timely and accurate diagnosis of MCAD is crucial for its effective management. Recent advancements in non-invasive imaging techniques have significantly improved the diagnostic accuracy for MCAD. High-resolution magnetic resonance imaging (HRMRI) and three-dimensional rotational angiography (3D-RA) have emerged as valuable tools in the diagnosis of MCAD [2,3].

HRMRI, particularly when combined with vessel wall imaging (VWI), allows for detailed visualization of the arterial wall, enabling the identification of key features such as the intimal flap, double lumen, and intramural hematoma [2]. This detailed imaging helps differentiate MCAD from other causes of stroke and guides therapeutic decision-making. Additionally, HRMRI can assess the extent of the dissection and monitor changes over time, providing valuable information for long-term management. This approach is applicable for slow-progressing or mildly symptomatic patients.

The cone-beam CT technique, available now on all new angiographic machines, provides detailed images of the vascular anatomy and the extent of the dissection. This information is crucial for planning endovascular treatment strategies. This technique allows for precise visualization of the vessel lumen and the identification of any associated aneurysms or stenoses. 

## 5. Treatment of MCAD

The treatment of MCAD has evolved with the development of advanced endovascular techniques and pharmacotherapy. The primary goals of the treatment are to restore blood flow, maintain cerebral perfusion, prevent thromboembolic events, and reduce the risk of hemorrhagic complications.

Endovascular stenting has emerged as a promising therapeutic option for MCA dissections. Stents provide mechanical support to the dissected artery and prevent further expansion of the false lumen. This reduces the risk of luminal collapse. 

Antithrombogenic coated stents are designed to reduce the risk of thrombus formation within the stent by promoting endothelialization and inhibiting platelet aggregation. They release antithrombogenic agents, such as heparin or paclitaxel, which prevent platelet adhesion and thrombus formation. The use of these stents has shown reduced incidence of thromboembolic complications and better long-term vessel patency [3].

### Coated Stents

Two different types of coated stents are used in our daily practice for endovascular stenting of MCAD: Credo HEAL (Acandis, Pforzheim, Germany) and pEGASUS (Phenox, Bochum, Germany).

The CREDO Heal stent, developed by Acandis GmbH, is an advanced stent used primarily in the treatment of intracranial aneurysms, but it can be used for stenosis and dissection treatment as well. This stent is distinguished by its innovative HEAL (Hydrophilic Endoluminal Aneurysm Lining) technology, which significantly improves its hemocompatibility and reduces the risk of thromboembolic complications. 

The CREDO Heal stent is constructed from a self-expanding nitinol mesh, designed to provide optimal coverage and support within the vessel while promoting endothelialization and reducing thrombogenicity. The stent’s structure ensures a high degree of flexibility and conformability, allowing it to adapt to the intricate anatomy of intracranial vessels [9].

HEAL technology is a proprietary surface modification that applies a fibrin-based nano-coating to the stent. This coating mimics the final step of the hemostasis process, promoting endothelial cell proliferation and reducing platelet adhesion. The fibrin mesh is further functionalized with covalently attached heparin, enhancing its antithrombogenic properties [10,11].

The biocompatibility of the CREDO Heal stent has been extensively studied in preclinical and clinical settings. In vitro and in vivo studies have demonstrated that the fibrin-based coating significantly reduces platelet adhesion and activation compared to uncoated stents [12,13]. Key findings include the following:

Endothelialization: HEAL-coated stents showed complete endothelialization within 28 days of implantation in preclinical models, with no significant differences in neointima thickness compared to non-coated stents. Macrophage Response: The number of macrophages present in the vessel wall was significantly lower for HEAL-coated stents, indicating a reduced inflammatory response. Fibrin and Platelet Deposition: HEAL-coated stents exhibited significantly reduced fibrin and platelet deposition on the stent surface, which is crucial for maintaining long-term patency [14].

The primary application of the CREDO Heal stent is in the treatment of intracranial aneurysms, particularly those with complex and broad-necked configurations. It can also be used for arterial stenosis and arterial dissection stenting. Its advanced design and coating technology provide several clinical benefits:

Thrombosis Prevention: HEAL technology reduces the risk of in-stent thrombosis, a common complication with flow diverters, by minimizing platelet adhesion and promoting rapid endothelialization [10,11]. Reduced Need for Dual Antiplatelet Therapy (DAPT): Traditional flow diverters require prolonged DAPT to prevent thromboembolic events. The enhanced hemocompatibility of the CREDO Heal stent allows for a potential reduction in DAPT duration, lowering the risk of hemorrhagic complications [15].

Versatility in Treatment: The flexibility and conformability of the stent make it suitable for a wide range of aneurysm morphologies and locations within the intracranial vasculature [16].

Hemocompatibility: Studies have shown that the fibrin/heparin coating of the CREDO Heal stent results in lower activation of the coagulation cascade and platelet activation compared to other devices [17,18]. Inflammatory Response: The CREDO Heal stent induces a lower inflammatory response, as evidenced by reduced macrophage infiltration and lower levels of PMN elastase [19]. Complement System Activation: The activation of the complement system, a critical factor in the body’s immune response to foreign materials, is significantly lower with the CREDO Heal stent [20].

The pEGASUS-HPC stent, developed by Phenox GmbH, is a novel device designed for the treatment of intracranial aneurysms and arterial stenoses. This stent is distinguished by its advanced Hydrophilic Polymer Coating (HPC), which significantly enhances its hemocompatibility and reduces the risk of thromboembolic complications. 

The pEGASUS-HPC stent is a laser-cut, self-expanding stent made from nitinol, a metal alloy known for its flexibility and shape memory. This design allows the stent to conform to the complex anatomy of intracranial vessels, providing optimal support while minimizing vessel injury [21]. The stent has an open-cell design, which enhances its ability to navigate through tortuous vascular pathways and facilitates precise deployment.

The HPC technology applied to the pEGASUS-HPC stent involves a multi-layered, glycan-based polymer coating that mimics the natural endothelial glycocalyx. This biomimetic surface reduces platelet adhesion and activation, thereby decreasing the risk of thrombus formation. The hydrophilic properties of the coating also promote rapid endothelialization, which is crucial for long-term vessel patency and reduced risk of in-stent restenosis [13,22].

Studies have shown that the HPC coating results in lower activation of the coagulation cascade and platelet activation compared to other devices [11,23]. The pEGASUS-HPC stent induces a lower inflammatory response, as evidenced by reduced macrophage infiltration and lower levels of PMN elastase [19]. The activation of the complement system, a critical factor in the body’s immune response to foreign materials, is significantly lower with the HPC-coated stent [20].

Preclinical and clinical studies have demonstrated the superior biocompatibility and hemocompatibility of the pEGASUS-HPC stent. In vitro studies have shown that the HPC coating significantly reduces platelet adhesion compared to uncoated stents. In vivo animal models have confirmed these findings, with HPC-coated stents exhibiting lower levels of thrombus formation and inflammation [13,24]. Additionally, the HPC coating has been shown to promote rapid endothelialization, with complete endothelial coverage observed within 28 days of implantation [14].

The primary clinical applications of the pEGASUS-HPC stent include the treatment of wide-neck intracranial aneurysms and symptomatic intracranial stenoses. Its advanced design and coating technology offer several clinical benefits. Unlike traditional stents that require dual antiplatelet therapy (DAPT) to prevent thromboembolic events, the pEGASUS-HPC stent’s enhanced hemocompatibility allows for the use of single antiplatelet therapy (SAPT) in selected cases, reducing the risk of hemorrhagic complications [25]. The flexible and conformable design of the stent makes it suitable for a wide range of aneurysm morphologies and stenosis locations within the intracranial vasculature [16].

## 6. Antiplatelet Management of MCAD

Pharmacotherapy plays a crucial role in the management of MCAD after stent placement. The choice of antiplatelet therapy is critical for balancing the goal of the prevention of thromboembolic events with the risk of hemorrhagic complications. Mono-antiplatelet therapy (MAPT) has gained attention as a potentially safer alternative to dual antiplatelet therapy (DAPT) in certain cases [26].

MAPT involves the use of a single antiplatelet agent, such as Clopidogrel, Ticagrelor, or Aspirin, to reduce the risk of thromboembolic events. This approach is particularly beneficial in patients with a high risk of hemorrhagic complications, as it reduces the overall bleeding risk compared to DAPT.

Cangrelor is an intravenous P2Y12 antagonist with a rapid onset of platelet inhibition. Due to its short half-life (3–6 min), it has to be administered in controlled continuous infusion with a set dosage per minute, which is calculated for a specific patient weight. The administration is started with an initial bolus dose, which is also calculated per kilogram of the patient’s weight.

After discontinuing the Cangrelor continuous infusion, platelet function returns to normal within 1 h. This rapid offset is very useful in case of bleeding complications and in situations that require surgery or other invasive procedures.

The above-mentioned pharmacokinetic features make this drug very useful in the antiplatelet management of acutely treated patients regardless of etiology that requires stent placement.

Another big advantage of Cangrelor is the possibility of a smooth transition to oral anti-aggregation with Ticagrelor. When performed properly, according to the protocol that we will discuss later, this bridging poses minimal risk of antiplatelet efficacy drop.

Moreover, according to available data, Cangrelor does not affect renal functions, and its effect is not modulated by age or sex [26].

Currently, there is no consensus in the neurovascular field about Cangrelor dosage, infusion duration, and bridging therapy. Protocols were built based on cardiology clinical trials. 

Ticagrelor is the drug of choice for bridging after Cangrelor is discontinued, as no significant interaction between these two drugs has been demonstrated. Ticagrelor can be administered during or after a Cangrelor infusion. Early administration of Ticagrelor (more than 1.25 h before stopping Cangrelor infusion) appears to modestly attenuate the increase in platelet reactivity during the first hour after discontinuation of Cangrelor and augments an apparent extent of platelet inhibition. Conversely, Clopidogrel may be unable to inhibit platelet aggregation and activation when administered concomitantly with Cangrelor [26].

A potential limitation of Cangrelor is the cost, which is higher than other available P2Y12 inhibitors. The possibility of short infusion therapy and early safe transition to long-term oral antiplatelet therapy may mitigate the economic burden. The safety demonstrated by a modified dose of Cangrelor in neuro interventions described by Aguilar-Salinas et al. can also decrease the cost of medication. Moreover, the real economic impact cannot be drawn until periprocedural complications and long-term patient outcomes are taken into consideration, with a possible tremendous upside of the short offset of the Cangrelor effect [26].

## 7. Materials and Methods

In preparation for this article, we reviewed all patients who were endovascularly treated in our department with a diagnosis of AIS in the anterior circulation from January 2022 until the end of March 2024. The treatments were performed at a comprehensive stroke center in Slovakia, Central Europe, which serves as an endovascular center for 13 primary stroke hospitals and a population of approximately 1.6 million people.

### 7.1. Patient Seleciton

During this period (January 2022–March 2024), we treated 548 patients diagnosed with anterior circulation stroke and large vessel occlusion (LVO) in our center. Among these, we identified 16 patients (2.92%) with a diagnosis of isolated MCAD. Nine of these patients were treated with antithrombotic coated stents. The remaining 7 patients received the non-coated self-expandable laser-cut CREDO stent with subsequent DAPT. For the purposes of this publication, we included both groups and compared their outcomes.

### 7.2. Endovascular Treatment of AIS

**Figure 1:**
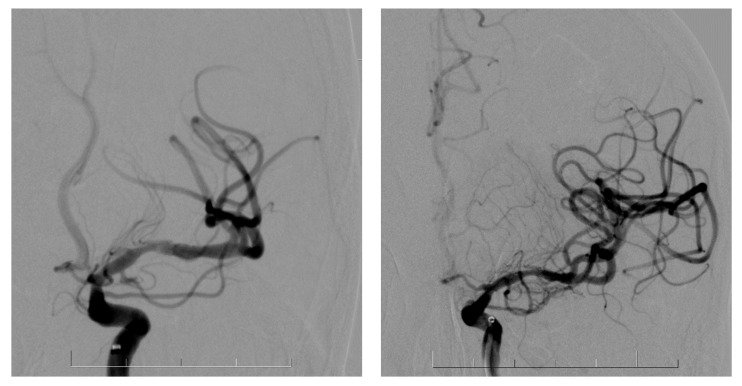
DSA with MCA dissection before and after the stent placement.

Endovascular treatment of anterior circulation AIS was performed in routine practice with a maximalist approach to achieve the best possible first-pass effect. We routinely proceeded with stenting after at least two good but unproductive passes of a mechanical thrombectomy or if vessel dissection was obvious on DSA or FD-CT (Figure 1).

### 7.3. Statistical Analysis

For statistical analysis of the results, we used the two-tailed Fisher exact test with an α-level of 0.05 for significance. The reason why we did not use the Pearson chi-square test was the small sample size. A multivariate model adjusted for potential confounders was not applied, which is the limitation of our statistical analysis. For the calculation of results, we used SPSS software (version 22.0; IBM, Chicago, IL, USA).

## 8. Technical Aspects of MCAD Management

The diagnosis of dissection was established during the endovascular procedure in our practice, based on either DSA appearance or 3D-RA images. After diagnosing the dissection, we typically waited and performed a delayed angiogram after 5 min to observe how the vessel responded post-recanalization. In cases of progressive occlusion, we conducted a non-contrast cone-beam CT (CBCT) directly in our angio suite to ensure there was no intracranial bleeding. If the CBCT was negative for hemorrhage, we proceeded with stent placement following appropriate antiplatelet preparation, which depended on the specific patient’s chronic medication. After stent placement, we performed control DSA runs to verify vessel patency. If the lumen was stable, we concluded the procedure and transported the patient to the neurological ICU for close monitoring.

### 8.1. Advanced Imaging during the Procedure

All data were acquired using a biplane flat panel detector angiography system (Artis zee biplane, Siemens Healthineers, Erlangen, Germany). FD-CT was performed using the commercially available image acquisition software Syngo VI20E DynaCT with an acquisition time of 20 s (20 s DynaCT Head, 70 kV). During acquisition, the C-arm with a 30 × 40 cm^2^ FD covered an angle of 200° with a 0.4° increment, assessing 496 projections with an image matrix of 1240 × 960 elements by using 2 × 2 binning of pixels. The system dose was set to 1.2 mGy/frame. A zoom format size of 22 cm was used. Post-processing of the FD-CT imaging image reconstruction was performed on a commercially available dedicated workstation (syngoXWP VD30B Workplace, Siemens) using the conventional and advanced reconstruction schemes by a third person not involved in the imaging analysis. Reconstructions were performed using Kernel type ‘HU’, image impression ‘smooth’, and a field of view of 18 cm. This resulted in a volume dataset with a batch of about 400 slices in a 512 × 512 matrix. Single slice thickness was 0.3 mm. The dataset was further processed as axial multiplanar reconstructions, adjusted to MSCT with a 5 mm slice thickness.

### 8.2. Antiplatelet Management during MCAD Treatment

For regular laser-cut stents, we most often used dual antiplatelet therapy (DAPT). The way of administering DAPT was dependent on the chronic medication of a specific patient. A number of patients suffering from MCAD were already on some kind of APT, which modified our strategy. 

**Figure 2:**
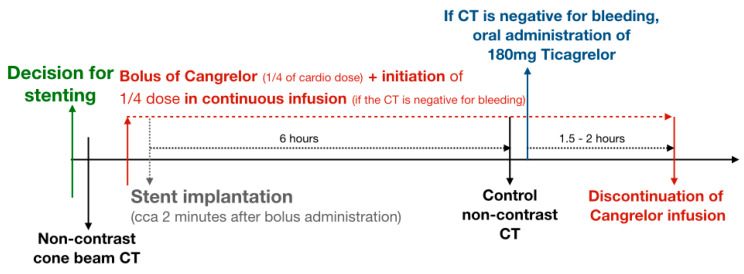
Scheme of reduced-dose Cangrelor usage with Ticagrelor bridging.

If the patient had not previously taken any APT, we administered intravenous (i.v.) ASA at a 300 mg dose plus i.v. Cangrelor. After six hours, we performed a conventional non-contrast CT (NCCT) to check for the presence of hemorrhage. If no bleeding was detected on the NCCT, we switched to oral ASA combined with oral Ticagrelor. However, with antithrombotic coated stents and no previous APT, we usually used mono-antiplatelet treatment (MAPT) with i.v. Cangrelor, followed by bridging to oral Ticagrelor (Figure 2).

In initial cases, we strictly guided our Cangrelor dosage according to the cardiology recommended dose, which led to frequent hemorrhagic complications, mainly in oropharyngeal, gastrointestinal, and urogenital tracts. In one early case, we encountered severe, symptomatic intracranial bleeding with the full cardiology dose, which led to the patient’s death.

Based on this experience, the dose of Cangrelor was established at ¼ of the cardiology dose by a gradual decrease.

To ensure sufficient effectiveness despite the reduced dose, we routinely measured platelet aggregability inhibition using the VerifyNow machine (Werfen, Bedford, MA, USA)—VerifyNow-P2Y12 Assay/VerifyNow PRU Test. The measurement was taken from venous blood, which was drawn at the end of each procedure. With a full cardiology dose, the platelet inhibition level was 99% or 100%. After lowering the dose to half cardiology, the inhibition remained at 95–99%. A 1/3 dose of Cangrelor still provided high inhibition with levels around 80–90%. Finally, when we applied 1/4 dose, the inhibition levels oscillated around 60–70%, which was sufficient for the prevention of ischemic events (stent thrombosis) and safe enough to diminish the risk of bleeding complications. 

## 9. Results

Between January 2022 and March 2024, we treated 16 patients with a diagnosis of MCAD. Seven patients received the bare metal, laser-cut, self-expandable CREDO stent with subsequent DAPT. After the introduction of coated stents, we started to use them in acute settings and nine MCAD patients received coated stents. Initial APT depended on the patient’s chronic medication.

Of the 16 MCAD patients (regardless of the type of stent), 7 required upfront mechanical thrombectomy, and 9 patients received standalone stenting of the MCA. 

The average age was 65.6 years, with the younger age group in the bare metal stent group (60.9 years). A lower average age was recorded in patients with worse clinical outcomes. The average NIHSS was 9.53 (4–19), with a median of 8. Five patients received intravenous thrombolysis (31.25%). Among the reasons for not administering i.v., r-tPA was most often recorded in an extended time window. Other reasons, such as chronic anticoagulation therapy, arterial hypertension not responding to medication, or fluctuating symptomatology, were recorded as well.

Successful TICI 3 recanalization with the first pass of mechanical thrombectomy was achieved in five out of seven patients. The remaining two patients received three mechanical thrombectomy passes, with three TICI results (Table 1 and Table 2).

In the bare metal stent group, the CREDO stent was used for all patients. Additional PTA was required in six out of seven patients. Intravenous Cangrelor use was necessary in three cases; four other patients were not eligible for Cangrelor because of upfront APT medication in the primary center, chronic APT, or chronic anticoagulation medication. Out of the seven DAPT patients, four encountered an intracranial hemorrhage HI1 or HI2, according to the ECASS II trial criteria (57.1%) [27], with no symptomatic intracranial hemorrhage (sICH) reported. The average NIHSS on the day of discharge was 6.4 [1,2,3,4,5,6,9,10,11,12,13,14,15,16,17,26,28], with an average NIHSS decline of 4.57. Four patients achieved good functional outcomes at 90 days, defined as mRS 0–2. One patient was mRS 4 at 3 months and two died (28.6%). The cause of death in one patient was extensive ischemic changes to the brain, and a second patient suffered stroke recurrence in a different brain territory despite DAPT. 

In the coated stent group, the CREDO Heal stent was used in five cases, and the pEGASUS stent was used in four cases. Additional PTA was performed in three cases. Intravenous Cangrelor with bridging to oral Ticagrelor was used in four coated stent cases. The other five patients did not receive Cangrelor for the same reasons as in the bare metal stents group. After the acute phase, patients continued with MAPT with oral Ticagrelor. None of these nine cases encountered intracranial hemorrhage. The average NIHSS on the day of discharge was 2.5 (range: 0–9), with an average NIHSS decline of 5.75. Seven patients achieved a good functional outcome of mRS 0–2, and two patients were at mRS 3 at the 90-day follow-up (Table 3).

After a careful review of the data, we discovered a statistical significance of non-symptomatic ICH occurrence after DAPT versus MAPT, with a *p*-value of 0.0192, which disproves the null hypothesis that different incidences of ICH in both groups could be coincidental. The multivariate model adjusted for potential confounders was not applied, which is the limitation of this finding.

**Figure 3:**
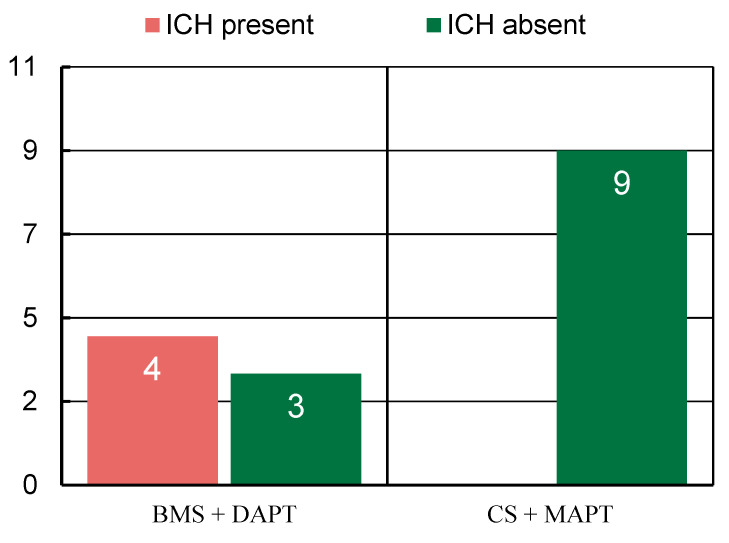
Graphic illustration of ICH incidence after stenting, comparison between coated and bare metal stent groups. ICH—Intracerebral Hemorrhage, BMS—bare metal stent, CS—coated stent, DAPT—dual antiplatelet therapy, MAPT—mono-antiplatelet therapy.

After using the same testing approach on other parameters, such as functional outcome, mortality, Cangrelor use, MT, or PTA, before stenting, none of these parameters showed statistical significance (Figure 3).

## 10. Limitations

Most of the available data on the use of Cangrelor to support stenting and subsequent APT management come from cardiology studies. Adjusting the dosage for neurovascular procedures requires a lot of clinical experience and good cooperation among clinicians.

Despite the good results in our center, the small sample size remains an important limitation. Larger cohorts of patients and multi-centric data are necessary to determine whether our findings are applicable in wider clinical practice.

Future research should aim to include a larger and more diverse patient population across multiple centers. This would enhance the generalizability of our findings and help to confirm our results in a broader demographic.

Ongoing research in our center focuses on long-term follow-up with a focus on functional independence of the patient and stent patency. These results should be available in the future, and our goal is to publish them to enhance the impact of our findings.

## 11. Conclusions

Isolated MCAD is a relatively rare cause of AIS in anterior circulation, but the natural history of the disease and the potential consequences of improper treatment make it very important to recognize. A better understanding of the disease and new diagnostic techniques have led to higher detection rates of this etiology in daily practice.

Ongoing research is focused on optimizing the use of advanced imaging techniques such as VWI, improving the design and efficacy of stents, and identifying the most effective pharmacotherapeutic regimens. 

The integration of antithrombogenic coated stents and MAPT represents a significant advancement in the treatment of this condition, offering the potential for improved outcomes and reduced complications such as ICH, which was supported in our small sample data as well.

In our experience, antithrombogenic coated stents are feasible and effective options for the treatment of these patients, even in an acute setting. The use of Ticagrelor MAPT with or without i.v. Cangrelor, followed by bridging to oral Ticagrelor, provides clinicians with a safe treatment option that has a good stent patency rate and a relatively low risk of hemorrhage compared to DAPT.

## Figures and Tables

**Table 1:** Demographic and baseline clinical characteristics of all patients, regardless of the type of stent: comparison between the patients with good and poor clinical outcomes after 90 days.

Parameter	mRS 0–2	mRS 3–6
*n* (%)	11 (68.8)	5 (31.2)
Males (%)	6 (54.5)	2 (40.0)
Age (mean)	71.3	54.2
Admission NIHSS (median)	8	15
Arterial hypertension (%)	9 (81.8)	4 (80.0)
Atrial fibrillation (%)	2 (18.2)	1 (20.0)
Diabetes mellitus (%)	4 (36.4)	2 (40.0)
Use of anticoagulation (%)	1 (9.1)	1 (20.0)
i.v. thrombolysis (%)	4 (36.4)	1 (20.0)

NIHSS—National Institute of Health Stroke Scale, i.v.—intravenous.

**Table 2:** Demographic and baseline clinical characteristics of all enrolled patients with data comparison based on the type of stent.

Parameter	Coated Stents and MAPT	Bare Metal Stents and DAPT
*n* (%)	9 (56.3)	7 (43.7)
Males (%)	5 (55.5)	3 (42.9)
Age (mean)	69.8	60.9
Admission NIHSS (median)	8	9
Arterial hypertension (%)	8 (88.9)	5 (71.4)
Atrial fibrillation (%)	2 (22.2)	1 (14.3)
Diabetes mellitus (%)	2 (22.2)	4 (57.1)
Use of anticoagulation (%)	1 (11.1)	1 (14.3)
i.v. thrombolysis (%)	4 (44.4)	1 (14.3)

NIHSS—National Institute of Health Stroke Scale, i.v.—intravenous.

**Table 3:** Selected parameters related to endovascular treatment and clinical outcome, and comparison between coated and bare metal stent groups.

Parameter	Coated Stents and MAPT *n* = 9	Bare Metal Stents and DAPT *n* = 7	*p* Value
MT before stenting (%)	5 (55.6)	2 (28.6)	1.0
PTA before stenting (%)	3 (33.3)	6 (85.7)	0.0601
i.v. Cangrelor required (%)	4 (44.4)	3 (42.9)	1.0
Onset to reperfusion time (median, min.)	283	332	
Any ICH after 24 h (%)	0 (0)	4 (57.1)	0.0192
NIHSS at discharge (median)	2	3	
NIHSS decline (median)	6	4	
mRS 0–2 at 90 days (%)	7 (77.8)	4 (57.1)	0.5769
mRS 3–6 at 90 days (%)	2 (22.2)	3 (42.9)	0.5962
90 days mortality (%)	0 (0)	2 (28.6)	0.175

MT—Mechanical Thrombectomy, PTA—Percutaneous Transluminal Angioplasty, i.v.—intravenous, ICH—Intracerebral Hemorrhage, NIHSS—National Institute of Health Stroke Scale, mRS—modified Rankin Scale.

## Data Availability

The datasets and images used and/or analyzed during the current study are available from the corresponding author upon reasonable request.

## Abbreviations

AIS	Acute ischemic stroke
ASA	Acetylsalicylic acid
BRIDGE trial	Bridging Anticoagulation in Patients who Require Temporary Interruption of Warfarin Therapy for an Elective Invasive Procedure or Surgery
CANTIC study	CANgrelor and Crushed TICagrelor in STEMI Patients Undergoing Primary Percutaneous Coronary Intervention
CBCT	Cone-beam computed tomography
DAPT	Dual antiplatelet therapy
DSA	Digital subtraction angiography
FABOLUS FASTER trial	Facilitation Through Aggrastat or Cangrelor Bolus and Infusion Over Prasugrel: a MUlticenter Randomized Open-label Trial in PatientS with STelevation Myocardial InFarction Referred for PrimAry PercutaneouS InTERvention
FD-CT	Flat detector computed tomography
GPI	Glycoprotein IIb/IIIa inhibitors
HEAL	Hydrophilic Endoluminal Aneurysm Lining
HPC	Hydrophilic polymer coating
HRMRI	High-resolution magnetic resonance imaging
ICU	Intensive care unit
i.v.	Intravenous
LVO	Large vessel occlusion
MCA	Middle Cerebral Artery
MCAD	Middle Cerebral Artery Dissections
MAPT	Mono-antiplatelet therapy
NCCT	Non-contrast computed tomography
NIHSS	National Institute of Health Stroke Scale
r-tPA	Recombinant Tissue Plasminogen Activator
SAH	Subarachnoid hemorrhage
TICI	Thrombolysis in cerebral infarction scale
VWI	Vessel wall imaging
3D-RA	Three-dimensional rotational angiography
